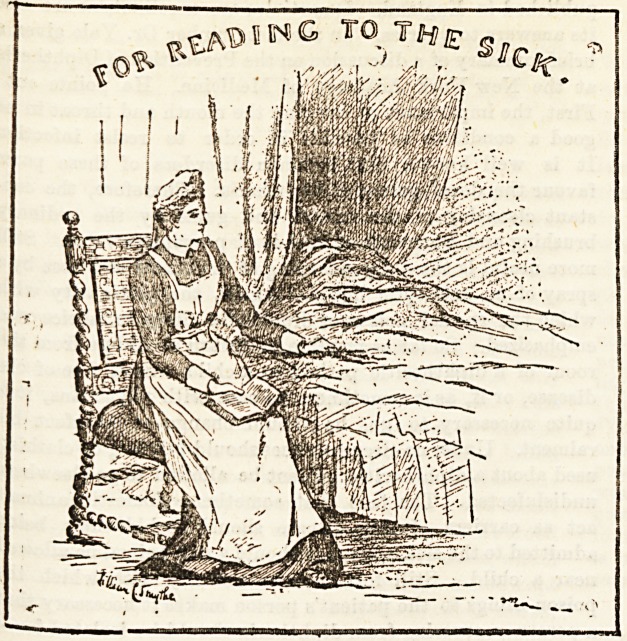# The Hospital Nursing Supplement

**Published:** 1891-08-15

**Authors:** 


### "The Hospital" Nursing Mirror.

Being the Extra Nursing Supplement of "The Hospital" Newspaper.

Contributions for this Supplement should be addressed to the Editor, The Hospital, 140, Strand, London, W.C., and should have the word "Nursing" plainly written in left-hand top corner of the envelope.

### En Passant.

NORWICH STAFF OF NURSES.—The report of the 25th year of work of the staff of hospital trained nurses at Bethel Street, Norwich, is very satisfactory. The demand for skilled nursing steadily increases, and 476 applications for nurses were received during the twelve months. Of these 346 cases were supplied, 61 of them being gratuitous, and many others at greatly reduced fees. The Committee would gladly increase the number of their nurses did their funds permit. Nine more nurses than the number now on the staff could be accommodated in their present house, and they would have been thankful to have had 40 nurses instead of 30 to meet the demands during the present year. Thanks are given to the nurses, and to the Lady Superintendent and the Secretary for their work during the last year.

MIDDLESBROUGH HOME.—On Saturday the Middlesbrough Amalgamated Cycling Clubs held a grand blossom and fancy costume parade, in aid of the District Nurses' Home in the town. The event drew together thousands of spectators, who, favoured with fine weather, enjoyed a spectacular exhibition, the like of which never before in the annals of Middlesbrough had been seen. About two o'clock the cyclists met upon the vacant ground behind the Town Hall, and in half-an-hour the open space presented quite a gay appearance, and as each character arrived they were greeted with shouts of applause, until at three o'clock fully a thousand riders had assembled. Some of the riders were attired in most fantastic costumes. Messrs. Fureson, of the Theatre Royal, having placed at the disposal of the cyclists their whole of the costumes, including those of the artists in the last Christmas pantomime, it may be surmised that the sight was well worth seeing. Several bands of music were present, and members of the mounted yeomanry and military cyclists were at the head of the procession, Dr. Cook and Mr. Dent in the scarlet coats of huntsmen. It would be difficult to give an account of the costumes, particularly as very few comical or conceivable costumes were omitted. Some of the notable characters were: The Zulu, the Sheeny man, with his box of glass and putty, Falstaff, a Shaker,several niggers, Moors, Spanish bullfighters, Slopers, griffins, crocodiles, &c. While the lady riders were all to be congratulated on their tasteful decorations, the Tandem Trike, bearing a flag, "Success to the Nurses' Home," ridden by two ladies, was very lovely, it being literally covered with flowers which, added to the pretty dresses of the riders, made it one of the most attractive displays of the procession. The gaily bedecked and merry processionists passed along the streets between dense rows of spectators, the collectors passing round the boxes for funds as they went. At the finish the whole of the riders filed up in columns in front of the Nurses' Home. The bands played several selections, finishing with the National Anthem, and hearty cheers from the public. In the evening a cyclists' night was held in the Theatre, when Mr. Caddick, Hon. Secretary to the parade, said he had much pleasure in announcing that upwards of £100 had been collected for the Nurses' Home. It was also decided to make the parade an annual benefit for the same.

SISTER FRANCES thankfully acknowledges the following: A large box of useful articles from the City Hospital, Edinburgh, collected by Nurse Gilchrist and Miss Dare, all new, and £2 in money; a cloak from the Matron of the Cottage Hospital, Stanmore; a beautiful handkerchief case filled and surgical scissors and books from Nurse Wilson, Bury St. Edmunds, together with 5s.; six new pinafores from Mrs. Goslett; from Miss Earle 5s.; St. Andrew's Vicarage, Bethnal Green, £1; Mrs. Homan, Netley Hospital, £1 4s.; Miss Crackenthorp, £1 4s., Leeds Nursing Home; Nurse Blackwell, one dozen linen aprons, Hampstead Infirmary, all being accompanied by letters of hearty good wishes; a parcel from Miss Proudfoot; collected by Major Johnson, £3 0s. 6d.; altogether collected through The Hospital, private friends, and the "Little Papers" appeal amounts to £60, and about £100 in goods.

UNIVERSITY NURSES.—One Saturday in July the nurses of University College Hospital were driven from London to Richmond in brakes, half the number leaving the hospital at eleven in the morning and the remainder at four o'clock. Dinner was provided at the Castle Assembly Rooms, and afterwards the nurses were taken for three hours on the river in boats provided by Sir Whittaker Ellis. At six o'clock the second batch of nurses arrived, and the whole number (64) sat down to tea. After tea the nurses were photographed in a group. The town band had been engaged to play on the lawn during tea, but only half the number turned up, half an hour late. The nurses who arrived last passed the rest of the evening on the river. The treat was organised by the Secretary of the hospital, Mr. Newton H. Dixon, to whom the nurses tendered their hearty thanks for a pleasant day. In the dusk of the summer's night the party drove back to town and duty, cheered and refreshed by their brief holiday.

THE PRINCESS OF WALES AND THE NURSES.—It has been decided that a screen containing the photographs of the first and second thousand nurses will be the most acceptable gift for presentation to the Princess of Wales, provided not less than 1,000 photographs are received. If the nurses will send their photographs (cartes preferred) and a postal order for 2s. to "Screen," Miss Pritchard, The Lodge, Porchester Square, London, W., by August 28th, they will be acknowledged each week in these pages. Nurses are requested to write their names and their policy number on the back of the photographs. If the 1,000 photos are not forthcoming, the money will be returned. Postal orders for 2s. each have been received from the following nurses: Nurses Kirk, Olive Jones, S. Love, C. Forrest, Stanton, Stacey, M. Hay, S. Bellamy, M. Trump, M. Kyte, Forster, H. Peck, H. Raven, S. Pennington, Walbank, M. Kershaw, Walker, Ovard, S. Little, M. Mackenzie, L. Wilshaw, E. Barkwith, E. Still, E. Burton, Lucie, A. Bridle, S. Openshaw, Speat, A. Speat, A. Macvicar, J. Hodgetts, E. Skelton, S. Richardson, A. Galbraith, A. Rags, C. Porter, L. Weatherby, C. Dearth, C. Cox, Ellis, R. Johnson, S. Philips, E. Stanton, M. Guernsey, G. Redit, A. Faulder, C. Walton, F. Grigg. Matrons, C. Mekin, M. Barnett, F. Ordish, A. Thompson, E. Whitworth.

### The Story of Sister Frances,

OF ST. LUKE'S HOME, VANCOUVER.

IN these days of sensational biographies and autobiographies it is quite refreshing to turn to the story of a noble career, simply and quietly pursued without any claim being made to the laurel wreath of fame. Sister Frances has told her story at our request, and we cannot do better than give the narrative in her own words. She says: Being left under rather distressing circumstances alone in the world, with a small income and no particular inclination towards a career of any kind, I went to the Rector of my parish for advice as to my future He persuaded me to enter St. Margaret's Home, Montreal to help the Sisters there until circumstances should lead me to a decision. Accordingly I left the North-West Territories where I had been living, and in following his advice had my first peep into charitable ministrations. The "Sisters" had only been in the Home a few weeks, and the internal arrangements being unsettled we all undertook what we could best perform, and my knowledge of housekeeping singled me out forthat branch of the work. Here I struggled and fought with expenses, learning the meanwhile the real meaning of the word poverty. All the time I was trying to settle what should be my ultimate work in life. The nursing profession seemed to embrace everything that interested me most. My rector approved my choice of a profession, and, with his sanction, I entered the Women's Hospital, Montreal, to begin my training. I had previously promised not to receive or touch my private means, and, therefore, I was forced to face drudgery, work, and training unknown in England. I was at this hospital six months, at the end of which time I received my certificates, and was also offered good appointments. Although I fully realised my inefficiency, I applied for the Matronship of the McGill and Montreal Maternity Hospital, and, being elected, began my work among the slums. I remained here working in connection with the Sisters of St. Margaret, under the direction of a Committee of ladies and the Rev. E. Wood. My residence here resulted in the opening of a Protestant Baby Home for the reception of illegitimate babies of the town, worked by the same Sisters, and also in the rebuilding and extension of my hospital, where I remained for two years. During this time I studied midwifery entirely. At the end of this period I had determined to become a volunteer missionary nurse in the North-West. Bishop Anson, of Qu'Appelle, who had taken a great interest in me, accepted me for work with him, and a fund was started in England for my future Cottage Hospital. Fate arranged that I should not take up my new work at once. Alas! a new Matron for the Maternity Hospital could not be found, and I had to remain till some one from England could be sent out. This caused a delay of six months, and I did not leave Montreal till September, 1887. By then the proposed scheme at Qu'Appelle had fallen through; the place was too poor to produce the necessary funds to establish a hospital, and the Bishop would not allow me to use my own resources. Soon after this frustration of my hopes the Rev. H. Fiennis Clinton arranged for me to proceed to Vancouver as a parochial nurse, and armed with credentials, introductions, and certificates, and with what I was much more proud of, my midwife's diploma, I landed in British Columbia. Before leaving Montreal, Mr. Wood admitted me to the Guild of St. Veronica, and for the first time I found myself wearing the badge of the Cross under which I have since worked.

When I got to my destination I found the place devastated by a recent fire, and nursing was almost an impossibility in the ruined houses. Acting upon my previous experience gained in St. Margaret's, I determined to open a similar Home in British Columbia. In six months I had the joy of finding my work sanctioned and my new big house complete and consecrated as St. Luke's Home. It was to be worked under the auspices of the Guild of St. Veronica. The establishing of this Home swallowed up the ready money that I could myself supply, or procure from other sources, so I had to make fresh efforts to increase the supply. Miss Hobbs, known as Sister Kate, of Guy's Hospital, came out to take charge of the general nursing, whilst I undertook the housekeeping and midwifery. Seven years of my life were thus passed without a rest of any kind. Then came the breakdown, and the present trip to England. Authorised by the Bishop, I have made a general appeal during my visit to this Island, which has resulted in about £150 in goods and money being entrusted to me for the benefit of the Home. Visitors are welcomed to St. Luke's, and last year we were visited by the Bishop of Qu'Appelle, Countess of Shrewsbury, and Lady Macdonald. Arrangements have been lately made at the Home for the reception of all mission workers going to Corea, and any such will receive a hearty welcome. Bishop Corfe, a personal friend of my family, made the Home his headquarters for the few days he was waiting for his ship. The Home is just now paying indoor expenses, my work being as yet quite voluntary. The work is under the superintendence of the Rector. The staff live a community life, and small salaries are given only to those who need, the training being gratuitous. The doctors freely help us in any way they can, both by tuition and voluntary services. The St. Luke's of to-day has a great future before it, and the present three workers have their whole hearts in their work.

### CONTENTMENT.—I.

"THE contented mind is a continual feast," says the old proverb, and the more we try for it the happier we shall be. There are some cheerful souls who can look on the bright side of life under almost any circumstances, but on inquiry they are generally found to have an anchor of hope in the great sea before the throne of God. It is the healthy ones who can get on tolerably well by not thinking; they have their work or their play, either of which prevents their dwelling on disagreeables. But the sick and lonely are more tempted to discontent; they have so much time to brood over their troubles and so little to distract their attention from themselves; thus they think their lot is miserable, their friends neglectful, their sufferings unbearable, their surroundings everything but what they would choose. Then they show their feelings in complainings and murmurings; they are very difficult to please, and are always trying to get their circumstances altered, so at last they fall into a state of selfishness which refuses to take an interest in people and things beyond themselves. Some folks show this spirit by words, and try to excite sympathy by drawing attention to their state, while others only put on a miserable look and dreary tone of voice; both kinds think everyone hard-hearted who does not give them their whole attention.

Is this a right state of things? It cannot be. Misery there is certainly, for discontented spirits are ever seeking rest and finding none. Sin doubtless there is also. The remedy for this lies within the reach of every sick person, but they must apply it for themselves, and earnestly cry to God to give them strength, and courage, and patience, and perseverance to apply it faithfully and unweariedly. The remedy is contentment, but there are a great many ingredients which go to make it up. The first is a difficult one to get; we must see and believe we are discontented, or we shall not realize the necessity of gaining it. Then having got thus far, we will force ourselves to remember it is sinful. We will not allow ourselves to make excuses for our behaviour; we will hide nothing from ourselves, but say, "I am discontented and will try to fight against it," and will at once begin with some small thing which is the most obvious to ourselves. It is a holy war we are engaged in, remember; we cannot fight alone, but if we daily and earnestly ask God's help He will help us. We may make but slow progress at first, yet with every battle the difficulties grow smaller, for the victory is of God.

Our friends have been far more patient with us than we have given them credit for. They have tried to please us and make us happy, but could not succeed. We have wearied their spirits and estranged our own hearts from them. No wonder we are unhappy; we are in a truly pitiable state. We must ask ourselves what shuts us from the love of friends and leaves us so sad and lonely. We think our lot a hard one, but the whole disposing is of the Lord, and we have no right to rebel against what our Creator sends. He shapes the pitcher with His wheel for His own ends and for our good.

"If thou wilt trust, thy Master will sustain;
And as thy days thy strength shall be."

This is the promise given to God's children; cast, therefore, thy burden upon the Lord, and He will bring it to pass.

### Nurses and their Ways.

WE have been spending a few weeks lately at a health resort, which appears to be a magnet for nurses, for there they most do congregate.

This charming town is within a reasonable distance from London, has an exhilirating air, and save from the hills, which surround it on either side, but the one that slopes down to the sea, is a perfect paradise for invalids. Invalids naturally bring nurses in their train, whom we see demurely walking by the side of Bath chairs, in which repose their patients or following with a more airy tread when a relation has for a season taken their post. We meet nurses on duty in uniforms of all sorts of sombre hues, from the rude specimen just evolved from the gamp germ, whose mortified black gown and white night cap with strings pinned on the back of her head, are hidden by a friendly cloak and veil, to the thoroughly trained nurse in her comfortable dark blue cloak and bonnet and neat shoes, whose attractive appearance makes one almost long for an occasion in which one might be nursed by her.

But many of the nurses we meet are on pleasure bent, simply taking a holiday to recruit their strength, and very thoroughly they seem to enjoy the needful rest.

Most striking of these is the butterfly nurse in her slate grey costume, with long veil of the same hue fluttering on the breeze as she rushes past us like the May Queen "in a flash of light." Demonstrative is she and voluble, unable to separate herself from her work under any circumstances, discussing with her companion in public vehicles, without reserve and *à haut voix* the numerous duties which await her return, and on alighting the word "case" floats back to us as with rapid step she vanishes from our sight.

Again they come in brown cloaks and bonnets, in cloaks and bonnets of almost any colour except red, but always with white strings and veil of ample dimensions falling from the latter. All these uniforms are to be more or less understanded of the lay mind, but what it cannot grasp is the strange, shall we call them women? (not always young) who disport mongrel garments, wondrous, undefinable, who make a far off, in some cases, a very far off attempt at conformity to type. At a distance some look like widows in "mitigated grief," as we approach we murmur, deaconess perhaps; but at close quarters we are as uncertain of their status as when they first loomed on our sight.

But what shall be said of those who in an ordinary dress seem to imagine that a pair of white strings to their headgear, and a something which passes for a "fall" give a *câchet* to their appearance calculated to impose on a guileless public? Who can they be? Are they the Uhlans, the Free Lances of the profession, who have taken up nursing after all other ventures have failed, and go about seeking whom they may devour? Alas! for those who fall into their hands. They forget, but the friends of the unoffending sufferer happily do not, that the question "where is your certificate?" can put such pretenders to the rout.

One sort of nurse and one only is conspicuous by her absence, and that is the religious nursing sister, but we understand she usually carries her placid cheerful face in the autumn to "gallant little Wales" and takes her modest outing at that season of the year.

To nurses one and all whether on duty or off we wish God-speed.

### American News.

The 5th annual report of St. Luke's Training School for Nurses at Chicago is just out. This is one of the best schools, running on modern lines, and ready with innovations; the Lady Superintendent is Miss K. L. Lett. There are now thirty-three graduates, six of whom are married, nine fill positions in hospitals, seventeen are nursing in Chicago, one is a physician, three are medical students; the remainder are at home, or still in St. Luke's Hospital. A District Nurse was sent out for the first time this year. The course of training is for two years, and the following particulars, culled from the rules, will be interesting to your readers:—

"Those wishing to obtain this course of instruction must apply to the Superintendent of the Training School, upon whose approval they will be received into the School for one month on probation. The acceptable age for candidates is from twenty-one to thirty-one years. The applicant should send with answers to the paper of questions a letter from a clergyman, testifying to her moral good character, and from a physician, stating that she is in sound health. Applicants are received at any time during the year when there is a vacancy. During the month of trial, and previous to obtaining a position in the School, the applicant must be prepared for an examination in reading, penmanship, simple arithmetic and English dictation. The examination is to test the applicant's ability to read aloud well, to write legibly and accurately, to keep simple accounts and to take notes of lectures. This amount of education is *indispensable* for a member of the School, but applicants are reminded that women of superior education and cultivation, when equally qualified as nurses, will be preferred to those who do not possess these advantages. The Superintendent has full power to decide as to their fitness for the work and the propriety of retaining or dismissing them. She can also, with the approval of the Board of Directors, discharge pupil nurses at any time, in case of misconduct or inefficiency. During the month of probation the pupils are boarded and lodged at the expense of the School, but receive no other compensation. They are not expected to wear the uniform of the School, but must come provided with dresses of washing material for use in the Hospital. They will reside in the Home, and serve for the first year as assistants in the wards of the Hospital; the second year they will be expected to perform any duty assigned them by the Superintendent, either to act as nurses in the Hospital, or to be sent to private cases among the rich or poor. The pay for the first year is $8 a month, for the second year, $12 a month. This sum is allowed for the uniform, text books, and other personal expenses of the nurse, and is nowise intended as wages, it being considered that the education given is a full equivalent for their services. They are required, after the month of probation, when on duty, to wear the dress prescribed by the Institution, which is of blue and white seersucker, simply made, white apron and cap, and linen collar. The day nurses are on duty from 8 a.m. to 8 p.m., with an hour off for dinner and additional time for exercise and rest. They are also often given an afternoon during the week, and have the right to the half of Sunday. A vacation of two weeks is allowed each year. All nurses are expected to attend the place of worship they prefer once on Sunday."

Two English nurses graduated last year, Miss E. Farrow and Miss A. Farrow; the former has been appointed Superintendent of Blessing Hospital, Quincy; the latter has been appointed assistant to Miss Lett.

The following is the statistical report of the John Hopkin's School, from its opening to the present time:—

| | | | |
|---|---|---|---|
| Total number of applications for school circulars | | ... | 625 |
| " " " " " refused | | ... | 206 |
| " " formal applications received | | ... | 137 |
| " " " " " refused | | ... | 105 |
| " " probationers received into school | | ... | 66 |
| " " accepted... | ... ... | ... | 48 |
| " " dropped during first year ... | ... | ... | 2 |
| " " remaining | ... ... | ... | 46 |

A magazine called *Babyhood* is in great request here amongst nurses in children's hospitals, and I believe it is also published in England. Its articles are excellent, as are also its answers to queries. In the last number Dr. Yale gives a brief summary of a discussion on the Prevention of Diphtheria at the New York Academy of Medicine. He points out: First, the importance of keeping the mouth and throat in as good a condition as possible in order to resist infection It is well known that common disorders of these parts favour the development of diphtheria. Therefore, the constant cleansing of the mouth and gums by the ordinary brushing and by mouth-washes is of preventive value. Still more useful is the daily cleansing of the throat and nose by a spray of an antiseptic liquid. Again, the pertinacity with which the special poison of diphtheria clings to fabrics was emphasized. So far as possible no person should go from the room of a diphtheritic patient to a child who is free of the disease, or if, as is sometimes the case with physicians, it is quite necessary to go, he should change or disinfect his raiment. Under no circumstances should bedding or clothing used about a diphtheritic patient be allowed to go elsewhere undisinfected. The fact that sometimes domestic animals act as carriers of this disease should forbid their being admitted to the sick room, nor should a sick animal be allowed near a child. Still further, the tenacity with which the poison clings to the patient's person makes it necessary that a person recovering from diphtheria should be isolated for a month at least.

An interesting article appeared in *The Nightingale* for July 4th. The writer had read Professor Billroth's "Care of the Sick," and so went to see over the Hospital at Vienna where he trains his nurses. A nurse there, as elsewhere, is required to have all the womanly virtues. Her training takes one year, including the month of probation. For this month she receives only board and lodging, from the beginning of the second month she receives in addition to this six guldens per month, also her clothing, including uniform, but excluding underwear. At the end of the first year the pupil undergoes an examination before the Superintendent and some members of the Board of Directors, which is conducted by the head physician. If she is successful, she receives the title of Nurse, receives the badge of the Society, and her salary is raised to twelve guldens per month. She receives no diploma as nurse, until she has occupied that position for one year. . . . . After being one year a pupil and one year a nurse her salary is raised to fifteen guldens per month, and after being one year a pupil and two years a nurse, she will receive the diploma of the "Sister of the Red Cross," (Rudolfiner) and a badge, which she has the right to wear on a white band on her arm. The nurses have no stated time off duty, and altogether though the arrangements seem rather better than at the big hospital at Vienna, they are poor compared to our training schools here, or to yours in England.

### Notes and Queries.

To Correspondents.—1. Questions or answers may be written on post-cards. 2. Advertisements in disguise are inadmissible. 3. In answering a query please quote the number. 4. A private answer can only be sent in urgent cases, and then a stamped addressed envelope must be enclosed. 5. Every communication must be accompanied by the writer's full name and address, not necessarily for publication. 6. Correspondents are requested to help their fellow nurses by answering such queries as they can.

### Queries.

(25).—How can I obtain admission as nurse in one of the naval hospitals? How am I to act, and to whom apply?

(26).—Will any nurse or doctor kindly tell me of a good book on the management of young babies.—*Nurse Browning.*

(27).—At what age are probationers taken at the London Hospital and at St. Bartholomew's?—*H. Barrington.*

(28).—Please supply me with professional address of some medical lady or gentleman (lady preferred) with whom I can correspond on a private matter. Can you also give me any idea of their fee for advice by private correspondence?—*L. M. E.*

### Everybody's Opinion.

[*Correspondence on all subjects is invited, but we cannot in any way be responsible for the opinions expressed by our correspondents. No communications can be entertained if the name and address of the correspondent is not given, or unless one side of the paper only be written on.*]

### DISTRICT NURSING.

"E.R." writes: I quite agree with the sentiments expressed by "An Old District Nurse." Sometimes certainly the nurse selected is not "the right person in the right place." I know from experience that district and hospital nursing are two very different things, but in the case I quoted the nurse was fully trained, and used to visiting the poor, and anxious and willing to do the work conscientiously, but she had too much spirit to work under an ignorant woman, who would only entrust her nurse with a ten oz. bottle of 1-40 carbolic. This quantity was supposed to be sufficient for a case of uterine cancer, five ulcerated legs, and other purposes. This lotion of course had to be added to warm water, so one can imagine the strength. The first day the nurse was given a two ounce ball syringe for the cancer case, and the superintendent stood at the foot of the bed to see that it was properly done, which it was to her satisfaction, except that the trained nurse kept the sheet over her patient. I am a duly certificated nurse, and once had the misfortune for a short time to work under one of the untrained Superintendents, who did not consider it a nurse's duty to obey a doctor, as the following will show. I was told to go and see a woman whose baby was ill. When I arrived at the cottage the mother said, "Nurse, the doctor says I am to rub the child back and front with camphor oil, and put on wool." I asked if she had done this and if she had plenty of both things. She replied in the affirmative, so I naturally asked what she wished me to do. Her reply astonished me, as she said, "I want your advice. The doctor says whatever I do I am not to poultice, but I think he is wrong, and I want to know if you don't think I best just put on a poultice." It is needless to say, I upheld the doctor. When I repeated this conversation to my all-wise "Lady Superintendent," she was highly indignant, and said, "If at any time you consider a doctor is wrong, you must change the treatment." This I said "I would not do." I ask my fellow nurses if any really properly-trained woman would dare to do such a thing? If nurses will not hold their own ground and positively refuse to work under an untrained Superintendent, then the evil will continue. Some women who have money and nothing to do, consider they are doing the right, and, in these days, fashionable thing, if they can go round with the district nurse, and to, "dabble" a little in nursing. The good these women do is far out weighed by the evil, if they are not really wise persons, and alas! few are.

### Appointments.

[It is requested that successful candidates will send a copy of their applications and testimonials, with date of election, to THE EDITOR, The Lodge, Porchester Square, W.]

MANCHESTER ROYAL INFIRMARY.—Miss Florence M. Calvert, Lady Superintendent, Monsall Fever Hospital (who was trained at Guy's), has been appointed Lady Superintendent. We congratulate Miss Calvert on her appointment, and hope she will find her new work in every way congenial.

HOSPITAL OF ST. CROSS, RUGBY.—Miss Barbara Chapman has succeeded Miss Petrie as Matron to the hospital of St. Cross, Rugby. Miss Chapman was trained at the Liverpool Royal Infirmary, where she had charge of the operation wards, then became Matron of the Llanelly Hospital, and for the last four years has been Matron of the St. John's Hospital, Weston Favell, Northampton.

### Christmas Competitions.

WE want none of our readers to go away for their holidays without taking some piece of work for wet days, which, when finished, can be sent to us for our Christmas parcels. So heartily were the garments for adults which we distributed last year appreciated, that we want this year to have twice the number. To encourage all to help us in this way, and to add interest to the work, we offer the following prizes, which will be awarded in books or money as the winners choose: (1) For the best pair of socks knitted by a nurse, 5s.; (2) for the best pair of socks knitted by any HOSPITAL reader, 5s.; (3) for the best made flannel shirt, 10s.; (4) for the best made woman's blouse, 10s.; (5) for the best made flannel petticoat, 10s.; (6) for the best made and best shaped dressing gown for an invalid cut out and made by a nurse, 20s. It will be seen that No. 1 and 6 are reserved for nurses only. With regard to No. 6 we specially hope for many entries, and if we secure them we propose to give more than one prize. Flannellette is cheap, and light, and warm, and would, therefore, form the best material for the dressing gown. In judging, four marks are given for workmanship, four for shape, and two for general appearance; therefore, it is not wise to spend time on elaborate trimmings. Long seams may be done by machine.

### The Princess of Wales' Fund for Mrs. Grimwood.

IN reply to correspondents, we have to state that any contributions Matrons, Sisters, and nurses desire to give to this fund, should be forwarded on or before the 18th inst., to Miss Knollys, Marlborough House, Pall Mall, London, S.W., when the lists will be finally closed. Nurse Spring's name was wrongly printed Spreng in last week's issue.

### Presentation.

MISS COLLINS, late charge nurse of the Huddersfield Infirmary, having resigned after a service of sixteen years, was, on the 11th inst., presented with a cheque of £30 by the Committee, also a very handsome travelling portmanteau and various other articles from the nurses by whom she was held in high esteem, a satchel from the patients as a token of respect, with every good wish for her prosperity.

### Amusements and Relaxation.

**SPECIAL NOTICE TO CORRESPONDENTS.**

**Third Quarterly Word Competition commenced July 4th, 1891, ends September 26th, 1891.**

Competitors can enter for *all* quarterly competitions, but no competitor can take more than *one first prize* or *two* prizes of any kind during the year.

The word for dissection for this, the SEVENTH week of the quarter, being

"WOODCOOK."

| Names. | Aug. 5th. | Totals. | Names. | Aug. 5th. | Totals. |
|---|---|---|---|---|---|
| Paignton | 28 | 206 | Christie | — | 44 |
| Psyche | 27 | 203 | Dulcamara | 27 | 197 |
| Hope | — | 47 | Nurse J. S. | 28 | 205 |
| Lightowlers | 28 | 215 | Qu'appelle | 28 | 208 |
| Wizard | 14 | 165 | E. M. S. | — | 68 |
| Wyameris | — | 46 | Jenny Wren | 21 | 188 |
| Dove | — | 62 | Carpe-diem | — | 65 |
| Punch | — | — | Grannie | — | 36 |
| Ivanhoe | 20 | 199 | Nurse G. P. | 15 | 138 |
| Tinie | — | — | Goodnight | — | — |
| Agamemnon | 28 | 212 | Gamp | 14 | 100 |
| Nurse Ellen | — | — | Charity | 21 | 85 |

**Notice to Correspondents.**

Jenny Wren, July 30th, 89; Qu'appelle 87.

All letters referring to this page which do not arrive at **140, Strand, London, W.C.**, by the *first post on Thursdays*, and are not addressed PRIZE EDITOR, will in future be disqualified and disregarded.

N.B.—Each paper must be signed by the author with his or her real name and address. A *nom de plume* may be added if the writer does not desire to be referred to by us by his real name. In the case of all prize-winners, however, the real name and address will be published.

### A Bad Quarter of an Hour.

["Two's company; three's trumpery."]

It happened in the Long. The drowsy heat of the July afternoon had departed, and as the shadows slanted, a delicious coolness stole in upon fagged bodies and wearied minds. There was no wind to speak of, and, therefore, it was a typical day, in the judgment of Dolly Strangways and her Sister Nell, for canoodling on the rippling reaches of the silver Thames.

The two girls were the daughters of one of the great luminaries of the ancient city of spires and towers; and both were nurses down from the hospital world for a holiday breath of home air and a spell of idleness.

"Isn't this ineffable?" cried Nell, as they paddled along, drinking in the summer loveliness of sky and river.

"It's not bad," rejoined Dolly, who was practical, and had no eyes to spare from her paddle. "Bob, you wretch," she went on, "sit still!"

The wretch addressed was an alert fox-terrier, on the incessant look-out for entertainment of some kind. Bob knew where he was, and his heart beat high as he eyed the river-banks; he guessed that his time was coming, and presently it came.

"Let's draw up alongside the bank," suggested Nell, and as they did so, she arranged herself on the folding lazy-back for a spell of dreamy rest. "Now, don't talk, Dolly, please! I'm going to have a good time."

"All right," said Dolly, "I shan't break the poetic trance. I'll amuse myself watching Bob, for evidently he's going to have a good time, too."

Bob had scrambled out of the canoe on to the bank, and was cautiously reconnoitring. If that bank were not a first-rate ratting ground, then Bob was not a thoroughbred terrier, with all his senses about him.

It was still and quiet; the river, unlike the busy water-world of a few weeks before, was well-nigh deserted. There was a faint rustle of grey aspens, a peaceful twitter of birds. So tranquil was the hour that Bob, trembling like a leaf in a gale, from sheer excitement, had the ecstacy of seeing his expectations realised. Cautiously stealing out of its hole came a good-sized rat, looking to the right and the left. Finding the coast clear—for Bob had won too many laurels at ratting to stir—as yet, the creature advanced until there was room for Bob to get in neatly behind it and its hole, and cut off escape. There was a sudden rush—a scrimmage; then a surprised exclamation from Dolly—"Why, Nell, I do believe Bob's seen a rat, he ——." That sentence was never finished, for on the peaceful evening air broke forth heart-rending shrieks from both the occupants of the canoe, shrieks that the stillness of the Long atmosphere might well have carried into the heart of the venerable city of learning.

In its frenzy to escape from Bob, and cut off from home, the rat desperately made for the stream, and darted into the canoe. Bob, in an equal frenzy, lost sight of where it had got refuge, and stood barking dolorously on the bank.

"Oh, Dolly, what is it?" Nell set up straight, her eyes wild with terror, but not knowing why she screamed.

"It's here!" gasped Dolly, white as a lily. "Didn't you see it spring in The rat! It's running about the bottom of the canoe. Help! murder! thieves!" and Dolly, gathering her skirts tightly together, screamed as loudly as a mezzo-soprano possibly could, stamping her feet by way of accompaniment in a reckless manner not usually adopted in such a crazy craft as a Canadian canoe.

"Oh, Dolly, come and hold me—never mind about yourself," moaned Nell in gasps, with a beautiful unselfishness, "I'm going to faint—to die right away!"

"Don't, for pity's sake, don't, or you'll capsize the canoe. Help—somebody! Ugh-h! Look at the brute!" and, sure enough, there was the rat peering up from their feet, in as abject a state of terror as themselves, and doubtless, if it could, it would have screamed as loudly as the girls.

"Shoo! shoo!" feebly cried Nell, who was still living; but the rat refused to be shooed.

"Don't do that!" shrieked Dolly. "You'll make it come on me, you heartless girl!"

"I'm *not* heartless; you are—I mean the monster is. Oh, Dolly, I *can't* stand it any longer. I'm going to put my feet over the side. I'll go mad if it touches them."

"No, no, you mustn't!" called back Dolly, who had thought of doing the same thing, but whose presence of mind was returning by degrees. "You'll drown us both, if you do. Listen. I'll sit still while you try to scramble out on the bank."

"Oh, I couldn't move," panted Nell, "I daren't loose hold of my skirts," for she, too, was under the impression that all the rat's energies were bent on attacking her knees.

Dolly drew a long breath. She had faced a few stiff sights and scenes, since she was a frightened slip of a probationer, but nothing that ever came near the awful horror of that small, brown quadruped scurrying backwards and forwards at the bottom of the canoe. "Its do or die!" she muttered, setting her teeth and squaring her shoulders. Seizing a paddle with one hand, while with the other she still clutched her skirts, she made a wild dash at the rat, and being a woman, missed it, precipitating herself on her face. How that canoe retained its equilibrium meanwhile, history does not say. Nell's screams were redoubled, but Dolly lay in a dumb, smothering agony, convinced that the rat was busy gnawing her nose and eyes, at least. One can die but once, was the wild thought of one who could calmly assist, and skilfully tend a brother man undergoing amputation of a leg. But then, a rat! The operating-room horrors paled before that living, creeping, squirming little monster, and Dolly said farewell to life, in a long-drawn sob. Overhead, Nell's voice sounded far-away in her ears. But not at first did its meaning penetrate her brain.

"It's gone, Dolly, the rat's gone! It jumped into the stream. We are saved!"

Dolly fearfully raised her face, one glance showed the blessed news was true. *Gr-r-r-r!* Sweeter than all earthly music was that sound as Bob worried the rat which he had neatly caught as it gained the bank. Scrambling up into position, regardless of the canoe's lurches, Dolly's first act was to carefully feel if her neat, little nose were still the distinguishing feature of her fair face. Next, the trembling sisters looked at each other, and then—they cried. After that relief, they felt better, and though so severely shaken that they could only wobble the canoe, somehow, home again, they did manage it; Bob, with all the pride of a victorious warrior, sitting in his old place between them.

Round the professor's dinner-table, that evening, the gruesome tale was told mid *ohs* and *ahs*, and scoffing jeers, according to the sex of the listeners. But not for many a night after did the two young nurses fail to dream harrowing dreams of "Three in a Boat"—themselves and that rat.

### Death in Our Ranks.

We regret to have to announce the death of Miss J. C. Jenkin ("Sister Catherine"), eldest daughter of Mr. W. S. Jenkin, of Liskeard, Cornwall, who died on Friday, July 31st, at the Royal Albert Hospital, Devonport, of typhoid fever. Miss Jenkin was trained at King's College, London, and afterwards worked in Bartholomew's. She was also one of the Sisters in the Royal Naval Hospital, Plymouth, for five years. She was most zealous in her work, and had a genial and kindly manner. Her death has cast quite a gloom over the Royal Albert Hospital.

## Figures and Tables

**Figure f1:**
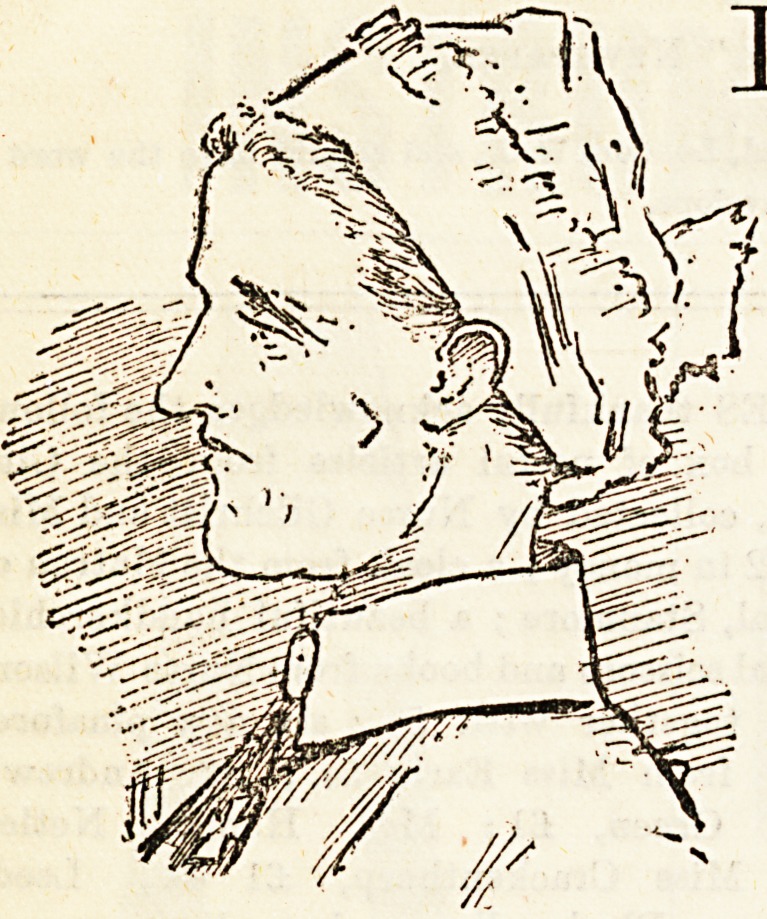


**Figure f2:**
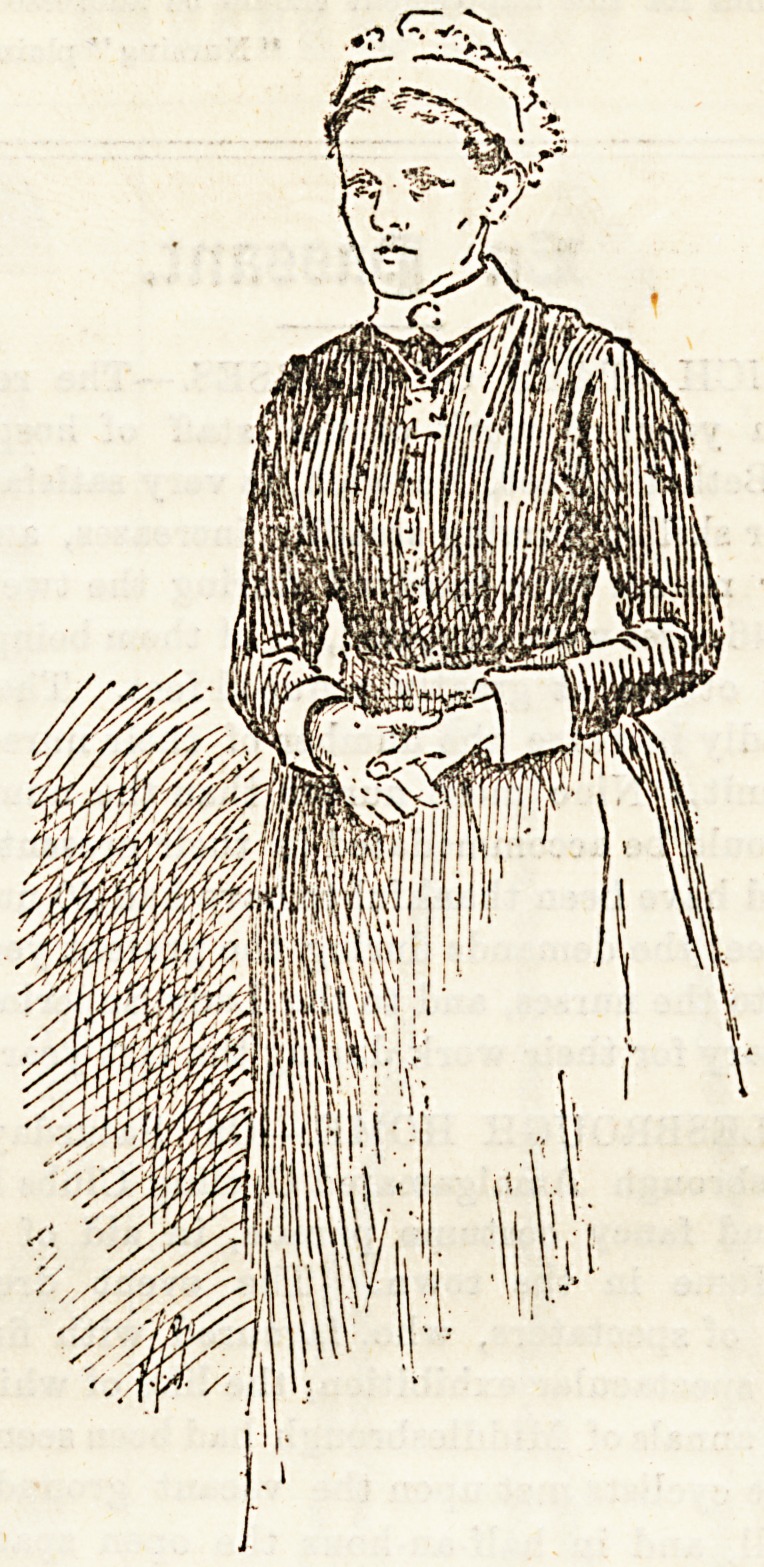


**Figure f3:**